# A Diagnostic Sign of Tuberculous Meningitis

**Published:** 1884-05-20

**Authors:** E. Van Goltdsnoven

**Affiliations:** Georgia


### Southern Medical Record:

EDITORS:

THOMAS S. POWELL, M.D. R. C. WORD, M.D.

*R. C. WORD, M.D., Managing Editor.*

☞All Communications and Letters on Business connected with the RECORD must be addressed to the Managing Editor.

VOL. XIV. ATLANTA, GA., MAY 20, 1884. NO. 5.

### ORIGINAL AND SELECTED ARTICLES.

### A DIAGNOSTIC SIGN OF TUBERCULOUS MENINGITIS.

BY E. VAN GOITDSNOVEN, M. D., OF GEORGIA.

Translated from the Paris Journal de Medicine.

Dr. Lambert Ott mentions as a sign of tuberculous meningitis, the excessive sensibility manifested by the patient upon pressure of the femur. He accidentally noticed this fact in a particular case and verified its presence in another; pressure on other points of the extremities producing no pain.

The mention of this clinical incident, clipped from the Philadelphia Medical Times, and which we copy from the Paris Medical, brings to our recollection a lecture of Professor Lasegue, in which he insisted upon the utility to be derived therefrom, and cited three remarkable facts, clearly bringing this symptom to light.

The first instance was that of a girl presenting a few symptoms peculiar to meningitis, which subsequently proved to be the case. In this Professor Lasegue accidentally noticed, whilst inspecting the cutaneous surface, an extreme sensibility of the femoral region. Indeed, the patient would utter a cry of anguish as soon as pressure was made, thus eliciting on the femur manifestations of a considerable hyperæsthesia which existed nowhere else.

With another patient this phenomenon was less marked, but pain existed in the same point of the femur. Complaint was also made of shooting pains in the lower extremities.

The third case was that of a girl of twenty, down with fever, accompanied by violent and delirious cephalalgia. This sign was well marked and defined. For the patient, though in a semi-comatose condition, briskly rose on her bed as pressure was made on her thigh.

The occasion which caused Professor Lasegue to recall these episodes of his practice, was the clinical treatment of a servant girl engaged in his household, and who, being afflicted with deafness, prostration and stupor, without either intestinal or thoracic troubles, presented the phenomenon in question in the highest degree.

No sooner was palpation commenced than she uttered a cry of anguish very different from that which bespeaks muscular rhumatism; for, in the latter, the pain is felt at a point perpendicular to the attachments and not to the bodies of muscles.

Facts of this order have been sounded by Professor Fournier who dwells on them in a special manner in his work on Cerebral Syphilis. For in his remarks on the congestive form of cerebral syphilis, after having described certain perturbations of general sensibility, such as formications or torpor generally detected in the extremities, Professor Fournier points out as disorders of a very different character, certain vague and indefinite pains, being produced on a limited part of the body, on a limb, on the extremities of a limb,pains sometimes fixed, sometimes movable, neuralgiaform, more or less persistent, and, in all cases, apt to recur.

What are those pains ?

Where is their true and precise seat ?

We are not able to tell.

Owing to the indecision which seems to shroud their localities, hardly any cognizance has been taken thereof. Hence they have received no other qualifications but those of Rheumatismal, Rheumatic, Neuralgic, Osteocopic Pains.

Professor Fournier says he has observed them so often (and always in the same condition), that he cannot but speak positively of their close relation to cerebral affections.

It cannot be denied that such pains are frequently produced as prodromic and symptomatic prenomena of cerebral lesions. Hardly noticed hitherto, they should be the object of closer investigation, as they constitute a symptom capable of furnishing a valuable clue in Diagnosis.

Professor Fournier also cites an instance which might be considered as closely allied to the above stated facts.

The case is that of a young man who had an attack of encephalo-meningitis. This disease, which developed rapidly and terminated fatally, was characterized by the pains in question in their highest intensity. The patient treated by Professors Fournier, Charcot and Edward Labbe, manifested such agonizing sufferings over the right femur, that repeated efforts were made to ascertain whether independently of cerebral affection, there was no particular lesion, such as a deep abscess, or periostitis, osteitis, etc. The closest and minutest examination failed to reveal the least cause for such a supposition, or to sustain the theory that anything of the kind could possibly exist ; and thus the localization and pathology of the pain remained unexplained.

With a view of calling attention to this particular form of pathological condition, Professor Fournier proposes to designate them as Cerebral Pains of the Limbs, with the understanding of modifying the term as soon as we shall be better informed and enlightened on their true pathology.

